# Low pH Enhances the Glucosinolate-Mediated Yellowing of Takuan-zuke under Low Salt Conditions

**DOI:** 10.3390/foods9111524

**Published:** 2020-10-23

**Authors:** Taito Kobayashi, Kei Kumakura, Asaka Takahashi, Hiroki Matsuoka

**Affiliations:** 1 Department of Health and Nutrition, Takasaki University of Health and Welfare, 37-1 Nakaorui-machi, Takasaki-shi, Gunma 370-0033, Japan; kobayashi-tai@takasaki-u.ac.jp (T.K.); kumakura@takasaki-u.ac.jp (K.K.); 2 Department of Nutritional Sciences, Tohto University, 4-2-7 Kamishima-cho Nishi, Fukaya-shi, Saitama 366-0052, Japan; asaka.takahashi@tohto.ac.jp

**Keywords:** pickled vegetables, yellowing salted radish root, glucosinolate–myrosinase system, tryptophan biosynthesis, isothiocyanates

## Abstract

This study was performed to clarify the enhancement of the 4-methylthio-3-butenyl isothiocyanate induced yellowing of salted radish root (takuan-zuke) by low pH during short-term salt-aging at low temperature and low salinity. We used two different methods to prepare the dehydrated daikon prior to salt-aging: air-drying outdoors (hoshi takuan-zuke) or salting with a stone press (shio-oshi takuan-zuke). Low salt-aging at low temperature was carried out under pH control with citrate-phosphate buffer. The yellowing of both types of takuan-zuke was accelerated below pH 5, and the color of air-dried takuan-zuke was deeper than that of salt-pressed takuan-zuke. To elucidate this phenomenon, several previously reported yellowing-related compounds were analyzed by high-performance liquid chromatography. The result showed that the production of the primary pigment, 2-[3-(2-thioxopyrrolidin-3-ylidene)methyl]-tryptophan, was low compared with that in previous reports. Therefore, we suggest that an unknown pigment was generated through a previously unreported pathway.

## 1. Introduction

Takuan-zuke (salted radish root) is a popular and traditional Japanese processed food. Japanese radish roots (*Raphanus sativus* L.; daikon) are dehydrated by either air-drying outdoors (hoshi takuan-zuke) or salting with a stone press (shio-oshi takuan-zuke) before pickling. The dehydrated daikon radishes are then pickled in salt or salty rice bran for several months. The color of takuan-zuke is transformed from the white color of daikon to bright yellow during the long salt-aging process at ambient temperature [1]. However, the yellow color of takuan-zuke is easily photobleached by visible light and often fades when it is displayed in stores [2]. Recently, the color of commercial takuan-zuke, which is pre-pickled at low temperature and low salt, has been noted to be a dull yellow. Commercial takuan-zuke is prepared using yellow coloring agents such as tartrazine and gardenia pigment for consistency. However, modern Japanese consumers prefer white takuan-zuke to yellow takuan-zuke, possibly due to the misunderstanding of why white radish is intentionally dyed yellow. The need for adding colorants can be avoided if the yellowing reaction is controlled.

**Figure 1:**
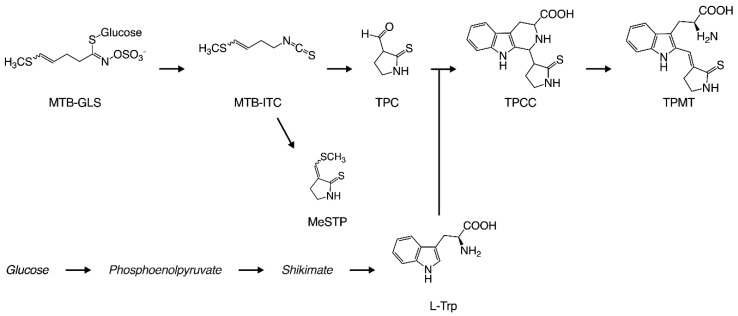
Yellow pigmentation process in takuan-zuke. Abbreviation. 4-methylthio-3-butenyl glucosinolate (MTB-GLS); 4-methylthio-3-butenyl isothiocyanate (MTB-ITC); 2-thioxo-3-pyrrolidinecarbaldehyde (TPC); 3-(methylthio) methylene-2-thioxopyrolidine (MeSTP); 1-(2-thioxopyrrolidin-3-yl)-1,2,3,4-tetrahydro-*β*-carboline-3-carboxylic acid (TPCC); 2-[3-(2-Thioxopyrrolidin-3-ylidene)methyl]-tryptophan (TPMT).

In our previous reports, we elucidated the detailed mechanisms of the yellowing reaction. Figure 1 shows the production process for the yellow pigment contained in takuan-zuke. Briefly, the starting compound for the yellowing reaction is 4-methylthio-3-butenyl isothiocyanate (MTB-ITC; raphasatin), which is the main pungent compound of radish. MTB-ITC is generated enzymatically from 4-methylthio-3-butenyl glucosinolate (MTB-GLS; glucoraphasatin), which is induced by cell damage during the dehydration process. Because MTB-ITC is readily degraded in the aqueous phase, it plays an essential role in the taste and flavor of processed radish [3]. MTB-ITC is converted to 2-thioxo-3-pyrrolidinecarbaldehyde (TPC) and 3-(methylthio) methylene-2-thioxopyrolidine (MeSTP), gaining a pyrrolidine ring by intramolecular cyclization and elimination of the methylthio group [4,5]. Furthermore, TPC, which has an aldehyde group, reacts with tryptophan via the Pictet–Spengler reaction to form 1-(2-thioxopyrrolidin-3-yl)-1,2,3,4-tetrahydro-*β*-carboline-3-carboxylic acid (TPCC) as a precursor to the yellow pigment [1,6]. 2-[3-(2-Thioxopyrrolidin-3-ylidene)methyl]-tryptophan (TPMT) from TPCC is the main yellow pigment in long-term salt-aged takuan-zuke [7]. The proportion of geometric isomers in TPMT is an important factor for yellow brightness and intensity, as the color of the (*Z*)-isomer at 100 ppm is significantly yellow with Δb* 10.5 compared to (*E*)-isomer. The *Z* to *E*-isomerization under visible light irradiation causes a chain reaction, making the preservation of the yellow color difficult without a light-shielding film [2]. We previously reported that TPMT formation from TPCC is a rate-limiting step that is pH- and temperature-dependent [7,8]. Therefore, it is difficult to use the characteristic natural color in the modern manufacturing method.

In our preliminary experiments, takuan-zuke was prepared with 10 mmol/kg buffering agent (pH 4, 5, and 6) at low temperature, to clarify the influence of pH on the yellowing reaction during the salt-aging process. We found that the yellow coloring during short-term salt-aging at low temperature was promoted under acidic conditions. Importantly, it is expected that microorganisms can be suppressed by pickling under acidic conditions [9]. In the present study, we aimed to evaluate the levels of known yellowing-related substances in takuan-zuke, and to understand the influence of acidic pH condition on the yellowing reaction for takuan-zuke.

## 2. Materials and Methods

### 2.1. Preparation of Yellowing-Related Substances from MTB-ITC

The yellowing-related substances, MeSTP, TPCC, and TPMT, from MTB-ITC were synthesized using previously reported methods [4,7,8,10]. MTB-ITC was synthesized by the reaction of a crude MTB-GLS solution extracted from radish sprouts, with myrosinase extracted from radish. TPC was synthesized by first dissolving MTB-ITC in acetone, adding acetic acid, and then sonicating the mixture. For synthesizing MeSTP, methanol and phosphoric acid were added to the MTB-ITC solution, and the mixture was heated under reflux (70 °C) overnight to obtain a precursor fraction. The precursor fraction was trans-solved in acetone, hydrochloric acid was added, and the mixture was synthesized by heating under reflux (60 °C). TPCC was synthesized by adding tryptophan and water to MTB-ITC, adjusting the pH to 2 or less with phosphoric acid, and sonicating. TPMT was synthesized by adjusting the crude TPCC solution to pH 7 and heating under reflux (37 °C). The synthesized compound was purified using column chromatography.

For preparing the internal standard for TPCC and TPMT analysis, 1-ethyl-1,2,3,4-tetrahydro-β-carboline-3-carboxylic acid (ETCA) was synthesized from L-tryptophan and propionaldehyde [11]. L-tryptophan (612 mg, 3.0 mmol) and propionaldehyde (191 mg, 3.3 mmol) were dissolved in 0.025 mol/L H_2_SO_4_ (26 mL) and allowed to react at 40 °C overnight. Next, a solid precipitate was collected. Subsequently, (*1S*, *3S*)- and (*1R*, *3S*)-ETCA were separated by preparative Octa Decyl Silyl (ODS) column using middle-pressure liquid chromatography (MPLC) (Smart Flash EPCLC AI-580S, YAMAZEN Co., Yodogawa-ku, Osaka, Japan) equipped with a Biotage^®^ SNAP Ultra C18 column (Filling amount 60 g; Biotage, Sweden).

To prepare the internal standard for MeSTP analysis, (*R*)-3-((*S*)-ethoxy(methylthio)methyl)-2-thioxopyrrolidine (EtOTP) was synthesized from MTB-ITC. MTB-ITC (2.0 mmol) was dissolved in the mixture of ethanol (80 mL) and 1 M phosphoric acid (20 mL). The mixture was concentrated under reduced pressure and extracted with ethyl acetate. The ethyl acetate extract was separated by preparative silica gel-MPLC system equipped with a universal column (Premium 2 L). Subsequently, (*S*)-EtOTP was separated by preparative ODS-MPLC system equipped with a SNAP Ultra C18 column.

### 2.2. Preparation of Salted Radish Roots (Takuan-zuke)

**Figure 2:**
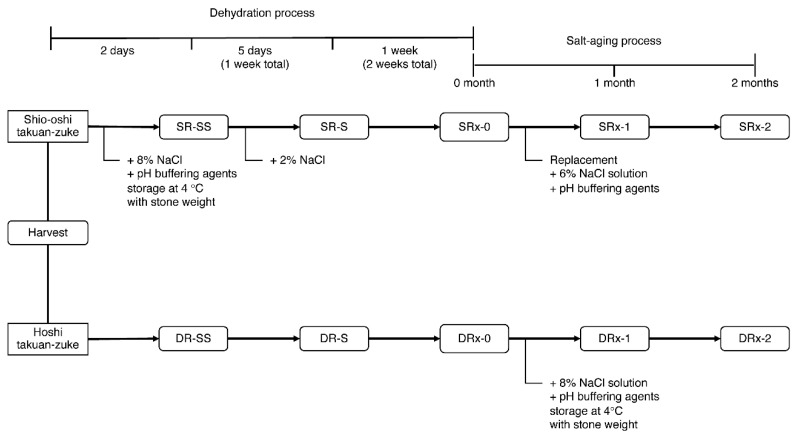
Time schedule for the pickling of daikon. SR indicates dehydration by salting (shio-oshi takuan-zuke). DR indicates dehydration by air-drying (hoshi takuan-zuke); SS indicates two days of dehydration; S indicates one week of dehydration. "X" is the buffer concentration, 0 mmol/kg (only NaCl addition) (SR0/DR0), 10 mmol/kg addition (SR10/DR10), 20 mmol/kg addition (SR20/DR20), and 40 mmol/kg addition (SR40/DR40).

Takuan-zuke was prepared according to a previously reported method with slight modification [8]. The pickling schedule is presented in Figure 2. In this experiment, we used the radish cultivar hoshi-riso daikon (Takii & Co., Kyoto, Japan), which was cultivated in a field at Misato-machi (Gunma, Japan) in August–November 2015. We prepared eight types of salted radish under different concentrations of McIlvaine buffering agent: 0 mmol/kg (only NaCl addition) (SR0/DR0), 10 mmol/kg addition (SR10/DR10), 20 mmol/kg addition (SR20/DR20), and 40 mmol/kg addition (SR40/DR40). The buffering agent was prepared using dipotassium hydrogen phosphate and citrate monohydrate (food additive grade, Kanto Chemical Co., Tokyo, Japan).

Hoshi takuan-zuke (abbreviated DR) was prepared according to the following method. After cutting off the root tips (3–4 cm), the whole daikon (269 kg including green leafy top) was hung to dry in a well-ventilated shady area for two weeks until it became dehydrated and flexible. After chopping the tops (3–4 cm) of root and the leaves, the hoshi daikon (78 kg) was pickled in 8 wt% NaCl (wet weight, the total weight of hoshi daikon and its priming water (33 wt% of daikon)) with pH buffering agents (0–40 mmol/kg of dehydrated daikon) for two months. The inner lid was placed on the daikon, and a stone weight (200 wt% of the dried daikon) was placed on the lid. The salting temperature was maintained at 4 °C.

Shio-oshi takuan-zuke (shio-oshi takuan-zuke, abbreviated SR) was prepared according to the following method. Fresh daikon (234 kg) was pickled with 8 wt% NaCl and 0−40 mmol/kg buffering agent of daikon and pressed under stone weights (200 wt% of daikon). After two days of salt-pressing, 2 wt% NaCl was added to the daikon, and the daikon was dehydrated for 12 days (total: two weeks). Shio-oshi daikon (27–35 kg) were pickled again in buffered saline (9−12 kg; 6 wt% NaCl, and 0−40 mmol/kg buffering agents). The pickling conditions (stone weight and storage temperature) were the same as that for hoshi takuan-zuke.

For tryptophan analysis, three types of dehydrated radishes (DR: air-dried daikon with leaves; nDR: air-dried daikon without leaves; SR: salt-pressed daikon without leaves) were produced by dehydrating them for one week using radishes that were collected in 2016. The dehydration process was conducted using the same procedure that was used for the study conducted in 2015.

For the sample, one barrel was prepared for each condition, and two bodies were collected according to the schedule shown in Figure 2. The division and reduced samples were rapidly frozen with liquid N_2_ and lyophilized. The lyophilized sample was subjected to freeze grinding and homogenization using a Multi-Beads Shocker (MB901THW(S), Yasui Kikai Co., Osaka, Japan). Lyophilized samples were vacuum packed and then stored at −30 °C.

### 2.3. Determination of Moisture, Salt Content, and pH of Takuan-zuke

The water content was determined by subtracting the lyophilized weight from the wet weight. The salt content was measured using the coulometric titration method (SAT-210, TOA-DKK Co., Tokyo, Japan). The pH of takuan-zuke was measured by first adding pure water to the lyophilized sample to restore the wet weight, and then using a pH meter (HM-30, TOA-DKK Co., Tokyo, Japan).

### 2.4. Color Measurement of Takuan-zuke

Color measurement of takuan-zuke was conducted based on a previous report [8]. The colorimetric change was measured with a Minolta CM-3500d Spectrophotometer, with a D65 illuminant and a 10-degree observer. The Commission Internationale de l’Eclairage (CIE) 1976 L*, a*, and b* color scale values of takuan-zuke were obtained by reflectance color measurement (measurement area: 8 mm diameter). Each value was measured in three places on the skin side of the upper, middle, and lower part of the root.

### 2.5. Quantitative Analysis of MTB-GLS in Takuan-zuke

Determination of glucosinolate in takuan-zuke was performed as previously reported [12]. The lyophilized sample (ca. 100 mg) was extracted by 1.5 mL of 80% MeOH at 75 °C. After the extraction mixture was heated at 75 °C for 10 min, 0.5 μmol sinigrin (internal standard, I.S.) was added. The crude extracts were adsorbed on an anion exchange resin (DEAE Sephadex A-25, GE Healthcare, California, CA, USA) and treated with sulfatase to convert desulfoglucosinolate overnight. The eluate containing the desulfoglucosinolate was quantified by ODS high-performance liquid chromatography (HPLC).

Analytical ODS HPLC was performed with an Agilent 1200-1260 system with a Poroshell 120 EC-C18 (100 × 3.0 mm ø, 2.7 μm; Agilent Technologies, Santa Clara, CA, USA). The flow rate was set at 0.85 mL/min, and the column temperature was set to 35 °C. Elution was achieved using a gradient of two eluents: H_2_O as eluent A and acetonitrile as eluent B. The gradient program was set at 0.2% B for 0.25 min, rising to 19.8% B at 6.00 min, and the remaining at 19.8% B to 7.00 min. Finally, the column was equilibrated using 0.2% B from 7.10 to 9.00 min. The results were detected at a wavelength of 229 nm.

### 2.6. Quantitative Analysis of TPC in Takuan-zuke

Quantification of TPC in takuan-zuke was performed by fluorescence derivatization method using 4-(*N,N*-dimethylaminosulfonyl)-7-hydrazino-2,1,3-benzoxadiazole (DBD-H) [13]. The lyophilized sample (10–50 mg) was added to 250 µL of 0.1% DBD-H in acetonitrile, 250 µL of 0.2 mM anisaldehyde in acetonitrile, and 500 µL of 0.5% trifluoroacetic acid in 60% (v/v) acetonitrile. The reaction mixture was shaken and incubated at 25 °C for 60 min. To 200 µL of the supernatant after centrifugation, 50 µL of 500 mM McIlvaine buffer (pH 5) and 50 mg of NaCl were added and shaken. The acetonitrile phase was considered the sample for HPLC.

Analytical HPLC was performed with an Agilent 1200–1260 system with a Poroshell HPH-C18 (100 × 3.0 mm ø, 2.7 μm; Agilent Technologies, Santa Clara, CA, USA). The flow rate was set at 0.85 mL/min and the column temperature was set to 40 °C. Elution was achieved using a gradient of two eluents: H_2_O as eluent A and acetonitrile as eluent B. The gradient program was: 25% B rising to 73% B at 5.5 min, further increasing to 100% B at 5.6 min, and remaining at 100% B to 5.99 min. Finally, the separation column was equilibrated using 25% B from 5.99 to 8.0 min. Fluorescence was detected with excitation at 450 nm and emission at 565 nm. Anisaldehyde was used as an internal standard.

### 2.7. Quantitative Analysis of Yellow Pigment-Related Substances in Takuan-zuke

The lyophilized sample (ca. 100 mg) was mixed with 250 µL of chloroform, 625 µL of methanol (including 40 nmol/mL EtOTP and 40 nmol/mL ETCA), and 250 µL of H_2_O (including 2.25% trifluoroacetic acid and 0.45% semicarbazide). The mixture was shaken and incubated at 37 °C for 30 min. After cooling on ice for 5 min, the mixture was centrifuged at 20,630× *g* for 1 min. To 1 mL of the supernatant, 500 µL chloroform and 500 µL H_2_O were added. After cooling on ice for 5 min, the mixture was centrifuged at 20,630× *g* for 1 min. The separated lower layer was obtained as a crude extract for MeSTP analysis, and the upper layer was obtained as a crude extract for L-tryptophan, TPCC, and TPMT analysis. The crude extract for MeSTP was concentrated to dryness using a centrifugal concentrator. The dried sample was dissolved in MeOH (300 µL) for HPLC analysis.

An aliquot of the upper layer extract was diluted to three times the original concentration, with 2% formic acid. A solid phase extraction (SPE) cartridge (Bond Elut Plexa PCX, 30 mg, 1 mL; Agilent Technologies, Santa Clara, CA, USA) was washed with 1 mL each of 1 M NaOH and 1 M HCl and conditioned with 1 mL each of MeOH and 2% formic acid. The diluted samples were loaded onto a PCX cartridge and washed with 1 mL each of 2% formic acid and methanol. The analytes were eluted with 1 mL each of alkaline eluent (30% ammonium hydroxide: 95% methanol = 5:95) in a tube containing 30 μL of concentrated formic acid. The eluate was evaporated to dryness with a centrifugal concentrator. After being dissolved in 200 μL of methanol, the sample solution was irradiated by long-wave ultraviolet (UV) light (UVGL-25, Funakoshi Co., Tokyo, Japan) at 375 nm and analyzed by HPLC.

Analytical HPLC was performed with an Agilent 1200–1260 system with a Poroshell HPH-C18 (100 × 3.0 mm ø, 2.7 μm; Agilent Technologies, Santa Clara, CA, USA). The flow rate was set at 0.7 mL/min, and the column temperature was set to 40 °C. Elution was achieved using a gradient of two eluents: 10 mM phosphate borate buffer (pH 8.2) as eluent A and methanol as eluent B. The gradient program was set at 15% B rising to 25% B at 7.00 min, rising to 100% B at 11.00 min. Finally, the column was equilibrated using 15% B from 11.01 to 13.00 min. The detection wavelengths were as follows: 268 nm for ETCA, EtOTP, and TPCC, 320 nm for MeSTP, and 400 nm for TPMT using a diode array detector. Tryptophan was detected by native fluorescence (excitation wavelength 285 nm, emission wavelength 348 nm). Each isomeric mixture of TPCC, TPMT, and MeSTP was separately quantified, and the results were documented as the sum. Internal standards were EtOTP for MeSTP and ETCA for the others.

### 2.8. Statistical Analysis

All quantitative data units are expressed in nmol per g of dry weight, and each value is expressed as the mean value ± standard deviation (*n* = 4). Multiple *t*-tests were performed using the Holm–Šidák method (α = 0.05). Significant differences between the treatment groups were determined with a two-way Analysis of variance (ANOVA), followed by a Tukey’s multiple comparison test using GraphPad Prism ver. 8 for Macintosh (GraphPad Software, Inc., CA, USA).

## 3. Results

### 3.1. Basic Data and pH Changes in Takuan-zuke Induced by the pH Buffering Agent

**Figure 3:**
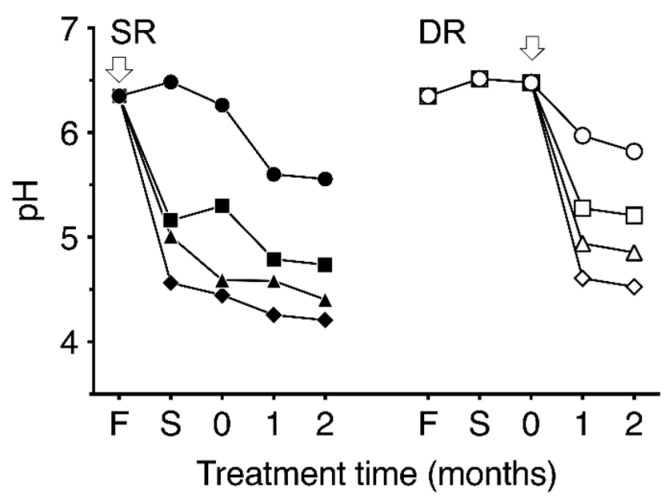
Time-dependent changes in pH during the dehydration and salting process with different concentrations of McIlvaine buffering agent. SR indicates dehydration by salting (shio-oshi takuan-zuke); DR indicates dehydration by sun-drying (hoshi takuan-zuke). The arrows denote the time point of salt addition. F denotes the pH obtained from fresh daikon. S denotes the pH obtained from the dehydrated daikon after one week of salting. “0” denotes the start of salt-aging process. Symbols refer to different concentrations of McIlvaine buffering agent (mmol/kg): ●, SR0; ■, SR10; ▲, SR20; ◆, SR40; ○, DR0; □, DR10; △, DR20; ◇, DR40. Values are mean ± standard deviation (SD) (*n* = 3). The error bar cannot be displayed because the standard deviation is small.

Temporal changes in pH in the two types of dehydrated daikon and takuan-zuke samples are shown in Figure 3. The pH of fresh daikon was 6.4. The pH of takuan-zuke without pH buffering agents gradually decreased to 5.6 in SR0 and 5.8 in DR0 after two months of salting. In contrast, the pH changes of SR- and DR-takuan-zuke with the addition of buffering agents were notably lower than in the case of non-buffered takuan-zuke. The pH values of the acidic buffered takuan-zuke samples decreased within one week of salting for SR groups and one month salting for DR groups. The pH lowering effect on DR samples by buffer addition was concentration-dependent, whereas no notable decrease in pH among SR samples was observed. With the addition of 40 mmol/kg buffer, the pH values of the SR40 and DR40 after two months of salting were 4.2 and 4.5, respectively.

### 3.2. Effect of Addition of pH Buffering Agent on the Color of Takuan-zuke

**Figure 4:**
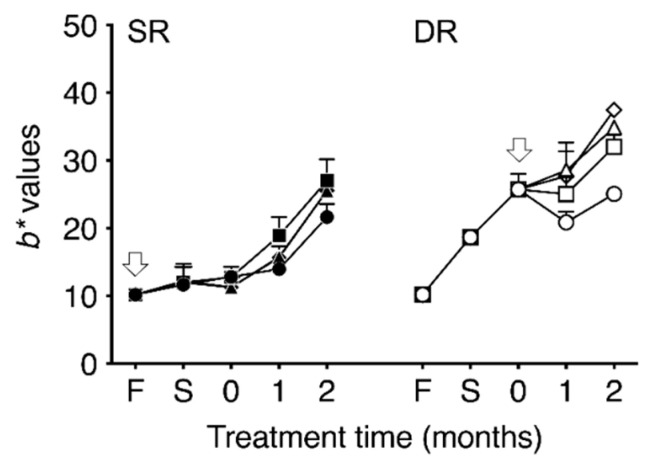
Time-dependent changes in b* values during the dehydration and salting process with different concentrations of McIlvaine buffering agent. SR indicates dehydration by salting (shio-oshi takuan-zuke); DR indicates dehydration by sun-drying (hoshi takuan-zuke). The arrows denote the time point of salt addition. F denotes the pH obtained from fresh daikon. S denotes the pH obtained from the dehydrated daikon after one week of salting. “0” denotes the start of the salt-aging process. Symbols refer to different concentrations of McIlvaine buffering agent (mmol/kg): ●, SR0; ■, SR10; ▲, SR20; ◆, SR40; ○, DR0; □, DR10; △, DR20; ◇, DR40. Values are mean ± standard deviation (SD) (*n* = 3).

In the fresh daikon, L*, a*, and b* values were 73.1 ± 4.0, −0.6 ± 0.2, and 10.2 ± 1.1, respectively. Although L* values for SR groups during salt-aging treatment fluctuated between 65.0 and 74.0, the difference that depended on buffer concentration was negligible. The a* values during salt-aging treatment changed in the negative direction. Figure 4 shows the b* values changes during the dehydration and salt-aging processes. Although b* value in the SR groups with buffered salting increased more than that in SR0, no buffer concentration-dependent changes in color were observed. However, the yellowing reactions in the DR groups were increased in proportion to the buffer strength. The b* value for DR groups by adding buffer increased significantly compared to that in the case for DR0. The ∆b* value of DR40 after two months salting based on fresh daikon was 2.4-fold that of SR0 and 1.8-fold that of DR0.

### 3.3. Effect of pH on the Yellow Pigment Production Pathway

**Figure 5:**
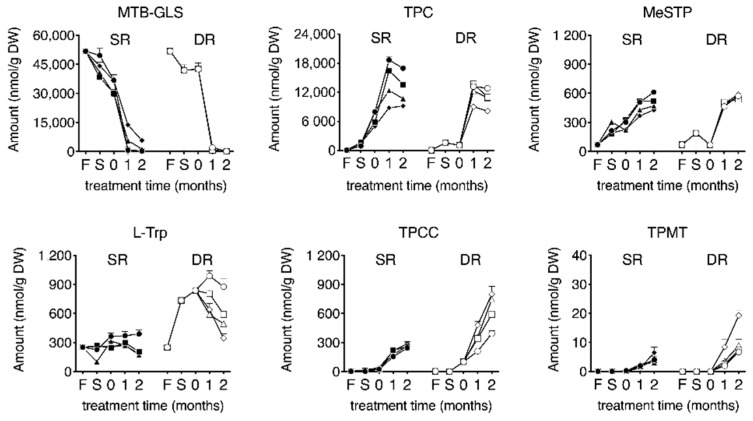
Change in the amount of each yellow pigment and related substances during the dehydrating and salting process. SR indicates dehydration by salting (shio-oshi takuan-zuke); DR indicates dehydration by sun-drying (hoshi takuan-zuke); F indicates the amount of each pigment obtained from fresh daikon; S indicates the amount of each pigment obtained from the dehydrated daikon after one week of salting. “0” time point indicates the start of salt-aging. Symbols refer to different concentrations of McIlvaine buffering agent (mmol/kg): ●, SR0; ■, SR10; ▲, SR20; ◆, SR40; ○, DR0; □, DR10; △, DR20; ◇, DR40. Values are mean ± standard deviation (SD) (*n* = 4). Data are analyzed using two-way Analysis of variance (ANOVA), followed by Tukey’s multiple comparison test. **Abbreviation.** 4-methylthio-3-butenyl glucosinolate (MTB-GLS); 4-methylthio-3-butenyl isothiocyanate (MTB-ITC); 2-thioxo-3-pyrrolidinecarbaldehyde (TPC); 3-(methylthio) methylene-2-thioxopyrolidine (MeSTP); 1-(2-thioxopyrrolidin-3-yl)-1,2,3,4-tetrahydro-*β*-carboline-3-carboxylic acid (TPCC); 2-[3-(2-Thioxopyrrolidin-3-ylidene)methyl]-tryptophan (TPMT).

The temporal changes in the yellowing-related substances in eight takuan-zuke samples are presented in Figure 5. The MTB-GLS level was highest at harvest (51.8 ± 1.0 μmol/g (dry weight; DW)). The levels of MTB-GLS during salt-pressing treatment were decreased and disappeared during the two months of salt-aging treatment. In the buffered SR samples, adding buffering agents slightly suppressed the degradation of MTB-GLS. The degradation rate of MTB-GLS in SR40 was slow compared to that in the other SR groups, and the residue rate in SR40 after two months of salt-aging was 11%. In the DR samples, slight hydrolysis of MTB-GLS was observed during the drying treatment; however, penetration of saline into dried daikon, during the salt-aging process, induced further hydrolysis of MTB-GLS.

MeSTP, as a degradation product of MTB-ITC, was generated from an early stage of salt and/or saline addition. The production levels were nearly equal between SR and DR takuan-zuke. The effect of buffer on MeSTP production was negligible.

TPC, the primary degradation product of MTB-ITC, was generated after salt and/or saline addition. TPC content in the SR groups reached maximum levels after one month of salt-aging (8.8–18.7 µmol/g). In the DR groups, TPC content increased with the addition of saline and reached a maximum after one month of salt-aging (9.0–13.7 µmol/g). TPC level decreased significantly after two months of salt-aging, depending on pH buffering strength.

The content of tryptophan in the fresh daikon was 255 ± 11 nmol/g. The analysis of tryptophan revealed that air-dried dehydration treatment resulted in a significant increase in its levels after harvest (hoshi processing for one week: 2.9-fold, *p* < 0.001; two weeks: 3.4-fold, *p* < 0.001). In contrast, shio-oshi treatment resulted in a slight change in the tryptophan content. Tryptophan in DR0 showed a maximum content value after one month of salt-aging (988 ± 52 nmol/g), and this value was 2.7 times higher than that in SR0 (*p* < 0.001). With subsequent salt-aging, tryptophan content significantly decreased, depending on pH buffering strength.

TPCC was generated immediately after salt and saline addition and increased with the duration of salt-aging after dehydration. The increased TPCC levels during the salt-aging process were significantly different between SR0 and DR0, and the content of TPCC after two months of salt-aging were 244 ± 8 nmol/g and 392 ± 31 nmol/g, respectively. The effect of pH on TPCC formation increased significantly with salt-aging time in the DR group, but no change was observed in the SR group. The TPCC levels in the buffered DR samples after two months of salt-aging were greater than that in DR0 (DR10: 1.5-fold, *p* < 0.001; DR20: 1.9-fold, *p* < 0.001; DR40: 2.0-fold, *p* < 0.001). Table 1 shows the changes in TPCC and tryptophan in the DR group; tryptophan, which is a substrate for TPCC, showed maximum content after one month of salting (Table 1). The decrease in tryptophan and the production of TPCC were almost equal in the buffered DR sample (DR10: 96%, DR20: 108%, and DR40: 91%).

The conversion of TPCC to TPMT started immediately after salt-aging and continued to increase after two months of salt-aging. The pH buffering strength significantly increased TPMT in the DR groups, with salt-aging time, whereas no effect was observed in the SR groups. The amount of TPMT was 6.7−19.3 nmol/g in the DR samples after two months of salting, which was 1.7–3.0 times greater than that in the SR samples.

### 3.4. Effect of Dehydration Method on L-tryptophan Metabolism

**Figure 6:**
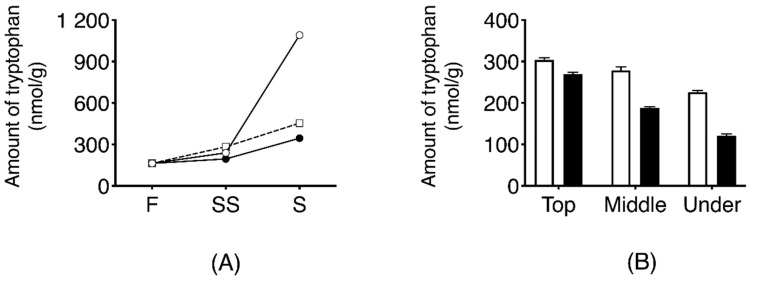
Temporal changes in the quantity of tryptophan obtained from daikon during the dehydrating and salt-aging process (**A**). Effect of dehydration treatment on tryptophan production. F indicates the value derived from fresh daikon. SS indicates the amount of tryptophan obtained from the dehydrated daikon after two days of salting. S indicates the amount of tryptophan collected from the dehydrated daikon after one week of salting. Symbols: ○, dehydrated by sun-drying with leaves (DR); □, dehydrated by sun-drying without leaves (nDR); ●, dehydrated by salt-pressing (SR). The error bar cannot be displayed because the standard deviation is small. (**B**). Localization of tryptophan in dehydrated daikon with leaves after two days of salting (DR-SS) white bar, inside radish; black bar, outside radish (on the skin).

Temporal change of L-tryptophan in three types of dehydrated daikon using those collected in 2016 (air-dried daikon with leaves; DR, air-dried daikon without leaves; nDR, and salt-pressed daikon without leaves; SR) is shown in Figure 6A. The content of L-tryptophan in DR samples was increased to 1.091 ± 13 nmol/g after seven days of drying, which was 6.7 times higher than that in fresh daikon. In the SR and nDR samples, the extent of increase of L-tryptophan was negligible compared to that in DR samples (*p* < 0.0001).

The localization of tryptophan in DR-SS is shown in Figure 6B. Tryptophan level was highest inside the upper part of the root at 304 ± 5.3 nmol/g. Tryptophan level inside the root was significantly higher than outside the root, and the levels reduced significantly toward the root tip.

## 4. Discussion

In this study, we analyzed the effect of acidic buffering agents on the yellowing reaction of short-term aged takuan-zuke. Air-dried radish (hoshi takuan-zuke) and salt-pressed radish (shio-oshi takuan-zuke), prepared under acidic conditions, were the brightest yellow at low temperature and low salinity. In particular, the b* value of hoshi takuan-zuke was equivalent to that of takuan-zuke, which was prepared by long-term salt-aging under room temperature and high salinity, as discussed in our previous report [8].

As described in Section 2, the pH buffering agent was added based on the wet weight of the daikon; the amount of buffering agent per radish, excluding water and salt, was 0.7 mmol/g (DW) for shio-oshi daikon, and 0.2 mmol/g (DW) for hoshi daikon. Therefore, the amount of buffering agent per dry weight affected the pH of takuan-zuke. There was no difference in the b* value of shio-oshi takuan-zuke at pH 5 or lower. Therefore, addition of 40 mmol/kg or more buffering agent into hoshi daikon did not affect yellowing.

To clarify the yellowing effects of acidic pH, the dynamics of the known yellowing-related substances were analyzed. In the takuan-zuke manufacturing process, in the absence of pH buffer, the conversion rate of MTB-GLS into TPC via MTB-ITC in the SR and DR samples was 36.1% and 25.5%, respectively. Since hoshi takuan-zuke showed a slight decrease in MTB-GLS during the drying process, the myrosinase reaction was thought to be suppressed under low water activity. During the salt-aging process, degradation of MTB-GLS was suppressed with increasing buffer strength, and the accumulation level of TPC was decreased. Myrosinase activity has been reported to peak at pH 5.7, whereas at pH 3.9, it decreases to 60% relative to the maximum activity level [14]. In addition, enzymatic reaction by myrosinase, under acidic conditions, has been reported to produce not only isothiocyanates but also nitriles [15,16,17]. Therefore, we suggested that the amount of MTB-ITC and TPC formed decreased due to the inhibition of enzymatic reaction or induction of nitrile formation.

Tryptophan content was significantly different between the two dehydration processes. Tryptophan levels in hoshi takuan-zuke increased from the air-drying dehydration process to early salt-aging. In hoshi takuan-zuke without leaves, no increase in tryptophan was observed, as with shio-oshi takuan-zuke. Tryptophan was seen to accumulate in the upper-inside part of hoshi daikon with leaves. Since tryptophan is reported to be synthesized by chloroplasts in plants [18,19], it was concluded that tryptophan is synthesized in the chloroplast of the daikon leaf and then transferred to the root through the vascular bundle. This result indicated that the increased tryptophan was synthesized in the chloroplasts of leaves during the drying process, rather than in microbial fermentation.

The content of TPCC, which is a yellow pigment precursor, was significantly increased in hoshi takuan-zuke than in shio-oshi takuan-zuke, similar to the results observed for tryptophan. In SR, the tryptophan production rate was the same as the reaction rate of TPCC synthesis, so it was inferred there was no apparent change in the tryptophan content. TPCC synthesis from tryptophan is considered a stoichiometric reaction when a sufficient amount of substrate is used, and tetrahydro-β-carboline synthesis via the Pictet–Spengler reaction has been reported to show high reaction efficiency under low pH conditions [11,20]. TPCC synthesis in takuan-zuke was revealed to be promoted at acidic pH, although the difference in tryptophan biosynthesis across the dehydration methods was a limiting factor for TPCC synthesis during the salt-aging process.

The content of TPMT in hoshi takuan-zuke was increased to a higher level than in shio-oshi takuan-zuke, similar to the results of tryptophan and TPCC. However, in this study, the conversion of TPCC to TPMT under low temperature and low salt conditions was very negligible. Our previous study had shown the optimal pH for TPMT synthesis from TPCC to be either weakly acidic or neutral pH in vitro [7]. In hoshi takuan-zuke at room temperature, the conversion rate to TPMT was 28.7%, and the color of salt-aged takuan-zuke varied to reddish yellow with prolonged salt-aging [8]. In the present study, there was no increase in the a* value despite the high b* value during the short-termed salt-aged process. These results suggested the contribution of TPMT to the yellowing of takuan-zuke to be low under acidic condition. In addition, HPLC analysis detected two characteristic peaks in takuan-zuke under acidic conditions. This peak had a maximum absorption wavelength of 392 nm and 404 nm, different from that in TPMT. It was suggested that these compounds are unknown yellow pigments that contribute to the yellowing reaction under acidic conditions.

In this report, we clarified that low pH conditions during takuan-zuke processing promotes an unknown bright-yellowing reaction at low temperature. In addition, we found TPC to be the most abundant among the known yellowing-related substances and an essential intermediate for takuan-zuke coloring. Imai et al. have reported that frozen and grated daikon, adjusted to pH 4 or below using acetic acid, turned yellow upon long-term freezing. This yellow pigment is generated by the condensation of two molecules of TPC [21]. Therefore, we proposed that an unknown yellowing reaction with MTB-ITC and TPC occurs during aging of takuan-zuke at low pH and low temperature. Future research would aim to elucidate the structure and reaction mechanism of the unknown pigment.

## 5. Conclusions

Temperature and pH conditions during salt-aging are the rate-limiting factors of the yellowing reaction, and we observed that takuan-zuke aged with low salt and at low temperature turns pale yellow. We found that the yellowing reaction was accelerated even at low temperature by the salt-aging of takuan-zuke under acidic conditions. The TPC level, which is one of the important intermediates of the yellow pigment, was highest after one month of salt-aging, regardless of the dehydration treatment. Tryptophan, another important intermediate, was increased only in dried daikon with leaves. The acidified and salt-aged treatment promoted the generation of TPCC, which is a pigment precursor. However, the generation of TPMT, which is a yellow pigment, was marginal compared to that in a previous report. Therefore, it was suggested that the unknown yellow pigment was generated via a pathway different from that described in the previous report regarding the yellow change of takuan-zuke under acidic and low temperature conditions. In future studies, it will be necessary to identify the unknown yellow pigment and the detailed mechanism underlying its generation.

## Figures and Tables

**Table 1:** Tryptophan and TPCC content, and their variation in hoshi takuan-zuke.

	Salt-Aging Time (months)	L-tryptophan				TPCC			
		DR0	DR10	DR20	DR40	DR0	DR10	DR20	DR40
Amount	1	988 ± 55	804 ± 39	588 ± 32	640 ± 63	210 ± 2	341 ± 24	357 ± 8	481 ± 36
nmol/g (DW)	2	876 ± 82	591 ± 22	494 ± 62	344 ± 46	392 ± 31	590 ± 4	745 ± 18	798 ± 81
Amount of change from DR0-1	1	-	−184	−400	−348	-	131	147	271
nmol/g (DW)	2	−112	−397	−494	−644	182	380	535	588

The amount of change in tryptophan and 1-(2-thioxopyrrolidin-3-yl)-1,2,3,4-tetrahydro-*β*-carboline-3-carboxylic acid (TPCC) is shown as an increase or decrease from DR0-1. Concentration of McIlvaine buffering agent: 0 mmol/kg (DR0), 10 mmol/kg (DR10), 20 mmol/kg (DR20), 40 mmol/kg (DR40). Data are expressed as means ± SD.
